# The Intracellularly Acting Effector Foa3 Suppresses Defense Responses When Infiltrated Into the Apoplast

**DOI:** 10.3389/fpls.2022.813181

**Published:** 2022-05-23

**Authors:** Nico Tintor, Gea A. M. Nieuweboer, Ilse A. W. Bakker, Frank L. W. Takken

**Affiliations:** Molecular Plant Pathology, Swammerdam Institute for Life Sciences, University of Amsterdam, Amsterdam, Netherlands

**Keywords:** PAMP, microbe, effector-triggered immunity, wilt disease, flg22, endocytosis, *Nicotiana benthamiana*, haustorium

## Abstract

Plant pathogens employ secreted proteins, among which are effectors, to manipulate and colonize their hosts. A large fraction of effectors is translocated into host cells, where they can suppress defense signaling. Bacterial pathogens directly inject effectors into host cells *via* the type three secretion system, but it is little understood how eukaryotic pathogens, such as fungi, accomplish this critical process and how their secreted effectors enter host cells. The root-infecting fungus *Fusarium oxysporum* (*Fo*) secrets numerous effectors into the extracellular space. Some of these, such as Foa3, function inside the plant cell to suppress host defenses. Here, we show that Foa3 suppresses pattern-triggered defense responses to the same extent when it is produced *in planta* irrespective of whether the protein carries the *PR1* secretory signal peptide or not. When a GFP-tagged Foa3 was targeted for secretion it localized, among other locations, to mobile subcellular structures of unknown identity. Furthermore, like the well-known cell penetrating peptide Arginine 9, Foa3 was found to deliver an orthotospovirus avirulence protein-derived peptide into the cytosol, resulting in the activation of the matching resistance protein. Finally, we show that infiltrating Foa3 into the apoplast results in strong suppression of the pattern-triggered immune responses, potentially indicating its uptake by the host cells in absence of a pathogen.

## Introduction

Plant pathogens are a major threat to agriculture causing significant yield losses. For successful colonization, pathogens need to manipulate their host, for example, by evading or suppressing its defense responses. Microbial pathogens, such as bacteria, fungi, or oomycetes, generally do this *via* a cocktail of secreted proteins, called effectors (Lo Presti et al., 2015; Uhse and Djamei, 2018; He et al., 2020). Plants on the other hand detect the presence of potential pathogens through a sophisticated surveillance system comprised of both cell-surface and intracellular receptors (Bentham et al., 2020; Zhou and Zhang, 2020). Detection of conserved structures that are typical for microbes, called pathogen- or microbe-associated molecular patterns (PAMPs or MAMPs) is mediated by transmembrane receptors. A well characterized example is flagellin (or its derived peptide flg22) that is recognized *via* FLS2, leading to a series of immune responses such as increased calcium influx, production of reactive oxygen species (ROS) and activation of mitogen-activated protein kinases (MAPKs; Defalco and Zipfel, 2021). Intracellularly localized immune receptors generally belong to the nucleotide binding and leucin-rich repeat containing proteins (NLRs) and detect race-specific effectors. Activation of NLRs results in a strong defense response, often culminating in localized cell death (referred to as hypersensitive response, HR; Yuan et al., 2021).

A significant portion of effectors is translocated into host cells, where they act as virulence or avirulence factors (Lo Presti et al., 2015; He et al., 2020). Whereas translocation of bacterial effectors *via* the type three secretion system is well established, it is little understood how fungal- or oomycete effectors reach the cytoplasm of host cells (Petre and Kamoun, 2014). Intracellular-acting oomycete effectors are frequently characterized by well-defined N-terminal amino acid motifs such as the RXLR or CRN sequence (Rehmany et al., 2005; Schornack et al., 2010). Initially, it was proposed that the RXLR motif mediates effector uptake by binding to specific phospholipids in the host cell membrane; however, recent data suggest that it rather plays a role during secretion into the extracellular space (Kale et al., 2010; Wawra et al., 2017). An intracellular function is shown for many fungal effectors; however, a shared amino acid motif is not apparent among them (Lo Presti et al., 2015). Prior to their internalization, *Magnaporthe oryzae* effectors localize to the biotrophic interfacial complex, a membrane-rich structure between the fungal hyphae and the plant cell membrane (Khang et al., 2010). Recently, a protein complex identified in *Ustilago maydis* was proposed to mediate translocation of effectors into the host cytosol (Ludwig et al., 2021). However, these structures have not been described in other plant pathogenic fungi. Thus, it remains a key question whether filamentous plant pathogens use special machineries to deliver effectors into host cells and/or whether certain effectors possess properties that allow them to pass the host cell membrane independent of a pathogen-encoded structure.

*Fusarium oxysporum* (*Fo*) is a widespread root-colonizing fungus, that can cause disease in more than 120 different plant species, many of which are agronomically important (Edel-Hermann and Lecomte, 2019). Individual strains carry partially overlapping effector sets, potentially determining their host range (Van Dam et al., 2016). Several *Fo* effectors were shown to act intracellularly as (a)virulence factors (Houterman et al., 2009; Di et al., 2017; Tintor et al., 2020). However, it remains unknown how they enter plant cells, since they do not share obvious motifs, or have common physio-chemical properties such as size or charge. Here we focus on Foa3, an effector of the Arabidopsis-infecting strain Fo5176 that inhibits both the flg22- and chitin-triggered ROS burst when expressed *in planta* without its endogenous signal peptide, indicating that it acts *via* an intracellular mechanism (Tintor et al., 2020). However, expression of full length Foa3 (e.g. including its signal peptide) resulted in a comparable suppression of both flg22- and chitin-triggered ROS accumulation, suggesting that the effector is taken up by the host cells. In this study we provide several lines of evidence pointing to translocation of Foa3 into the host cytosol when it is targeted for secretion or infiltrated into the apoplast.

## Materials and Methods

### Plasmid Construction

The constructs pCAMBIA-*35S::Sw-5b* and pCAMBIA-*35S::NS21-YFP* were described previously (Zhu et al., 2017). The signal peptide coding sequence of *Nicotiana tabacum PR1* was amplified from PVX::ECP1 (Laugé et al., 2000) and subcloned into pDONR201 and pGWB451. To generate *SPpr1-NS24-YFP*, the signal peptide sequence was PCR-amplified using primer FP8256/FP8257 and as template pDONR-SPpr1 (primers are listed in Table 1). Likewise, the *NS24-YFP* cassette was amplified using primer FP8258/FP8259 and the template pCAMBIA-*NS21-YFP* (Zhu et al., 2017). Primer FP8258 N-terminally introduced three additional amino acids to *NS21-YFP*, resulting in *NS24-YFP*. This was done to ensure that a stretch of hydrophilic amino acids follows the signal peptide and thus ensures proper cleavage by the plant secretion machinery. The amino acids were the same as in the full length NSm protein at that position. The *SPpr1* and *NS24-YFP* fragments were fused by overlapping PCR with primers FP8260/FP8259, thereby introducing an *Xba*I and a *BamH*I site. The obtained product was cloned into the *Xba*I/*BamH*I sites of pBIN61 (SLDB3104; (Tameling et al., 2010) and named pBIN-*SPpr1-NS24-YFP* (Supplementary Figure 1). To create *SPpr1-NS24-YFP-Arg9*, the *SPpr1-NS24-YFP* cassette was amplified by PCR using primers FP8260/FP8592 and pBIN-*SPpr1-NS24-YFP* as template. In a second PCR the restriction sites *Xba*I/*Xma*I were added by PCR using primers FP8260/FP8593. Finally, the obtained fragment was cloned into the *Xba*I/*Xma*I sites of pBIN61, resulting in pBIN-*SPpr1-NS24-YFP-Arg9*. To generate *SPpr1-NS24-Foa3*, the backbone of pBIN-*SPpr1-NS24-YFP* was amplified by PCR using primers FP8847/FP8848. The *Foa3* coding sequence (genbank ID: FOXB_16928) lacking the signal peptide sequence (*dsp* for deleted sp) was amplified from pBIN-*dspFoa3-HB* (Tintor et al., 2020) with primers FP8849/FP8850, thereby introducing overlaps with *NS24* and the vector backbone. Finally, these two fragments were fused using the In-Fusion technology, following the manufacturer’s instructions (New England Biolabs). The obtained construct was named pBIN-*SPpr1-NS24-Foa3*.

**Table 1:** Primers used in this study.

FP8256	ATGGGATTTGTTCTCTTTTCAC
FP8257	GGCACGGCAAGAGTGGGAT
FP8258	ATATCCCACTCTTGCCGTGCCAATAAGGTTATCAAGATCTGTCCA
FP8259	AAAGGATCCTCCTTACTTGTACAGCTCGTCC
FP8260	AATTCTAGAATGGGATTTGTTCTC
FP8592	CCTTCTACGACGTCGTCGCCTTCTTCTCTTGTACAGCTCGTCC
FP8593	AATCCCGGGCTACCTTCTACGACGTCG
FP8847	GGATCCTATCACAATCCTAG
FP8848	GGAGGATCCTACCCTTATGATG
FP8849	TAGGATTGTGATAGGATCCGTGGAATACAAGTGG
FP8850	TCATAAGGGTAGGATCCTCCAAATTTGCCACAACC
FP9155	AAAGCAGGCTCCATGGGATTTGTTCTCTT
FP9156	AAAGCAGGCTCCGTGGAATACAAGTGGGTT
FP9157	AGAAAGCTGGGTCAAATTTGCCACAACCCAG
FP872	GGGGACAAGTTTGTACAAAAAAGCAGGCT
FP873	GGGGACCACTTTGTACAAGAAAGCTGGGT
FP9627	CAAAAAAGCAGGCTTAATGGTGGAATACAAGTGG
FP9628	CCACTTGTATTCCACCATTAAGCCTGCTTTTTTG

To C-terminally fuse them to GFP, *dspFoa3* and *SPpr1-Foa3* were first subcloned into pDONR207. *dspFoa3* was amplified with primers FP9156/FP9157, and the Gateway adapters were added with primers FP872/FP873. The obtained fragment was subcloned into pDONR207 following the manufacturer’s instructions. The start codon was added by Quick Change using primers FP9627/FP9628. *SPpr1-Foa3* was amplified from a pBIN-*SPpr1-Foa3-NS24* construct by using primers FP9155/FP9157 and introduced into pDONR207. pGWB451-*dspFoa3-GFP* and pGWB451-*SPpr1-Foa3-GFP* were generated by Gateway recombination.

### Transient Transformation of *Nicotiana benthamiana*

*Agrobacterium tumefaciens* strain GV3101 was transformed with binary vectors and used to transiently transform 4–5-week-old *N. benthamiana* plants as described previously (Tintor et al., 2020). Plants were kept in a greenhouse at 20°C without artificial illumination. For Western blots, apoplastic fluid isolation, ROS and MAPK assays, Agrobacteria were infiltrated at an OD_600_ of 0.3. A strain containing the silencing suppressor P19 was co-infiltrated at the same OD_600_ (Tintor et al., 2020). For cell death assays and confocal microscopy, all Agrobacterium strains, including P19, were infiltrated at an OD_600_ of 0.1.

### Apoplastic Fluid Collection

Apoplastic fluid was isolated from *N. benthamiana* leaves 2–3 days after infiltration with Agrobacteria. To this end, deionized water (MQ) was infiltrated into leaves using a needleless syringe. Excess water was removed from the surface with filter paper. Next, the leaves were rolled up and placed into the outer unit of a 20-ml syringe, which was inserted into a 50 ml falcon tube. Apoplastic fluid was released by centrifugation at 1,000 g for 10 min at 4°C. Next, the apoplastic fluid was filtered through a PVDF membrane (0.45 μm pore size, low protein binding) to remove any Agrobacteria, and either used directly or stored at −20°C.

### ROS and MAPK Assays

Flg22-triggered ROS generation was monitored as described earlier (Felix et al., 1999). ROS production was induced with 200 nM flg22 and measured with help of a luminol/peroxidase solution [250 μM luminol (Sigma), 10 μg/ml peroxidase (Sigma)]. Apoplastic fluid derived from *N. benthamiana* plants that produced dspFo3, spFoa3 or spGFP was infiltrated into leaves of 4–5-week-old *N. benthamiana* plants and 3 h later leaf discs were excised and kept overnight on MQ. Flg22-triggered ROS production was measured the next morning.

Flg22-triggered MAPK activation was performed as described previously, with a few modifications (Flury et al., 2013). Three days after Agro-infiltration, *N. benthamiana* leaves were infiltrated with 0.5 μM flg22 or MQ and 10 min later leaf discs corresponding to 15 mg leaf material were collected and immediately frozen in liquid nitrogen. Total protein was isolated as described earlier and MAPK activation was monitored by Western blot using an anti-p44/p42 MAPK antibody (D13.14.4E; Cell Signaling Technology). Apoplastic fluid derived from *N. benthamiana* plants that produced dspFo3, spFoa3 or spGFP was infiltrated into leaves of 4–5-week-old *N. benthamiana* plants and 3 h later 1 μM flg22 or MQ was infiltrated. Ten minutes after flg22 treatment, leaf discs were collected, frozen in liquid nitrogen, and processed as described above.

### Protein Isolation and Western Blotting

Protein isolation and western blotting was carried out largely as reported before (Tintor et al., 2020). Briefly, soluble proteins were isolated from *N. benthamiana* by grinding 20 mg leaf tissue in 150 μl lysis buffer [50 mM Tris–HCl pH 7.5, 2% SDS, 10 mM DTT, 1x protease inhibitor cocktail (Roche), 1 mM PMSF] and centrifuging at 16,000 g for 10 min. Proteins were separated by SDS-PAGE, using 12% gels. As primary antibody monoclonal anti-GFP antibodies (ChromoTek) were used at 1:6,000 dilution. The secondary goat-anti-rat antibody (Pierce) was used at 1:8,000. The signal was visualized using the ECL Plus kit (GE Healthcare) according to the manufacturer’s instructions.

### Cell Death and Confocal Imaging

Cell death in *N. benthamiana* leaves was visualized similarly as described (Landeo Villanueva et al., 2021). Four days after *Agrobacterium* infiltration red fluorescence was visualized using a ChemiDoc imaging system (Universal Hood III, BioRad), using Qdot 605 filters and 30 s exposure time. Live cell imaging was carried out 2–3 days after Agro-infiltration using a Nikon A1 confocal microscope. GFP was excited at 488 nm with an Ar-ion laser, and emission was detected at 500–550 nm. RFP was excited at 561 nm using a dpss-laser and emission was detected at 570–620 nm. Images were captured using a 20x water-immersion objective or a 40x oil-immersion objective. Images were composed using Fiji software.

## Results

### Both dspFoa3 and SPpr1-Foa3 Inhibit PTI

**Figure 1:**
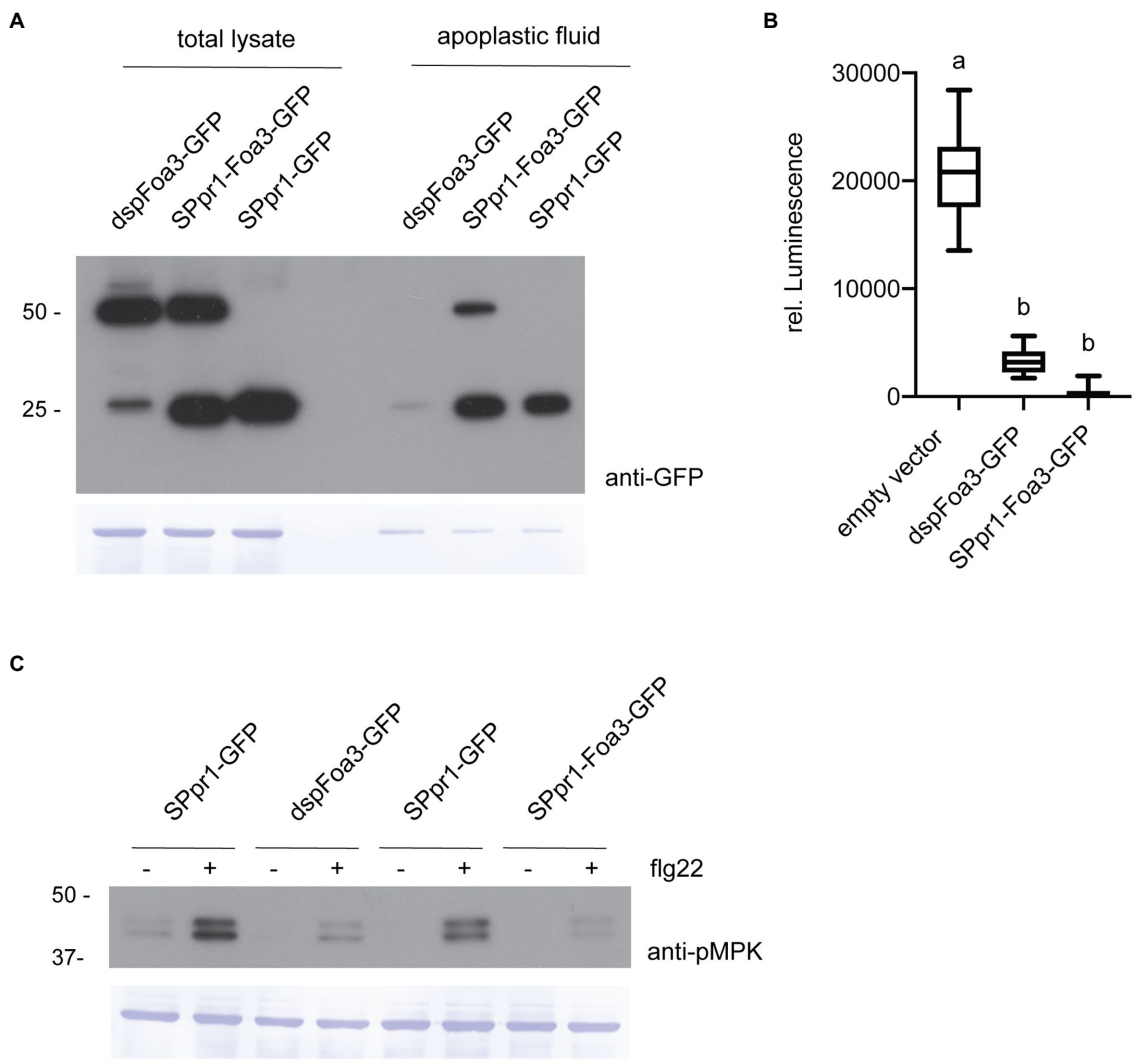
Expression of Foa3 with or without the PR1 signal peptide sequence suppresses flg22-triggered defense responses. **(A)** Anti-GFP western blots showing accumulation of dspFoa3-GFP, SPpr1-Foa3-GFP and SPpr1-GFP in total protein lysates and in apoplastic fluid of *Agrobacterium* infiltrated *Nicotiana benthamiana* leaves. Molecular weight (kDa) markers are shown on the left. Equal loading is verified by Coomassie staining of the blots. **(B)** Flg22-triggered ROS burst in *Nicotiana benthamiana* plants expressing the indicated constructs. Boxes extend from the 25^th^ to the 75^th^ percentile, whiskers from the lowest to highest values, bar indicates the median, *n* = 12 leaf discs. Significant differences are indicated by the different letters (*p* < 0.05, Kruskal-Wallace test). **(C)** Western blot showing flg22-triggered activation of MAP kinases in *Nicotiana benthamiana* leaves expressing the indicated constructs. Leaf discs were exposed to water (−) or flg22 (+) for 10 min. Molecular weight (kDa) markers are shown on the left. Equal loading is verified by Coomassie staining of the blots. Experiments were repeated three times with similar results.

We previously reported that a Foa3 version lacking its endogenous signal peptide (dspFoa3) suppressed flg22- and chitin-triggered ROS production, pointing to an intracellular virulence function (Tintor et al., 2020). Interestingly, expressing the full-length gene, thus encoding the signal peptide, resulted in a comparable level of ROS suppression. To exclude the possibility that the fungal Foa3 signal peptide is not efficiently recognized by the plant secretory system, we generated a construct where the signal peptide sequence derived from *Nicotiana tabacum* PR1 was fused to Foa3. This PR1 signal peptide was shown in previous studies to effectively target proteins for secretion (Hammond-Kosack et al., 1994; Laugé et al., 2000; Ziegler et al., 2000). In addition, both dspFoa3 and SPpr1-Foa3 were C-terminally tagged with GFP to allow their detection *via* Western blot and live cell imaging. To test whether the fusion proteins were produced to similar amounts *in planta*, both constructs were expressed in *N. benthamiana* using Agrobacterium mediated transient transformation. A construct encoding GFP fused to the PR1 signal peptide sequence was included as a control. Western blotting with anti-GFP antibodies revealed that dspFoa3-GFP migrated at around 50 kDa, which corresponds to the expected size of the dspFoa3-GFP fusion protein (Figure 1A). In contrast, for SPpr1-Foa3-GFP two bands were detected at roughly 25 and 50 kDa, indicating that the GFP tag was frequently cleaved off, but also that a significant amount of the GFP tag remained fused to the effector. Next, apoplastic fluid was collected to test for presence of secreted Foa3. The apoplastic fluid of leaves expressing *dspFoa3-GFP* revealed only a very weak GFP signal, indicating that in the absence of a signal peptide, this protein remains largely intracellular (Figure 1A). The residual amount detected in the apoplast could potentially originate from damaged cells. In contrast, both free GFP as well as the Foa3-GFP fusion protein were readily detected in the apoplastic fluid of *SPpr1-Foa3-GFP* expressing leaves (Figure 1A). These results show that dspFoa3 remains intracellular, but that adding the PR1 signal peptide results in its secretion into the apoplast.

Next, we wanted to test whether Foa3 could still exert its (intracellular) defense-suppressing activity after being targeted to the apoplast by the PR1 signal peptide. To test this, *N. benthamiana* leaves infiltrated with Agrobacteria strains containing, respectively, an empty vector, *dspFoa3-GFP* or *SPpr1-Foa3-GFP* were treated with the bacterial elicitor flg22. Subsequently, the production of ROS was measured over time. As expected, the empty vector-treated leaves showed high levels of ROS induction, whereas the presence of dspFoa3-GFP resulted in a strong suppression of this defense response upon flg22 application (Figure 1B). Importantly, the SPpr1-Foa3-GFP producing leaves showed a similar or even slightly stronger ROS suppression as compared to the dspFoa3-GFP leaves (Figure 1B). To corroborate this finding, a second defense output was tested, namely activation of MAP kinases by flg22. For this *N. benthamiana* leaves expressing either *spGFP* as control or *dspFoa3-GFP* or *SPpr1-Foa3-GFP* were mock-treated or infiltrated with flg22. The activation of MAP kinases was monitored by Western blot using an antibody that recognizes phosphorylated MAP kinases. Whereas the *spGFP*-expressing leaves showed a strong accumulation of phosphorylated MAPKs, presence of dspFoa3-GFP or SPpr1-Foa3-GFP resulted in an almost complete suppression of this defense response (Figure 1C). These findings show that even though the effector was targeted for secretion, Foa3 still efficiently suppressed PTI responses.

### Subcellular Localization of Foa3

**Figure 2:**
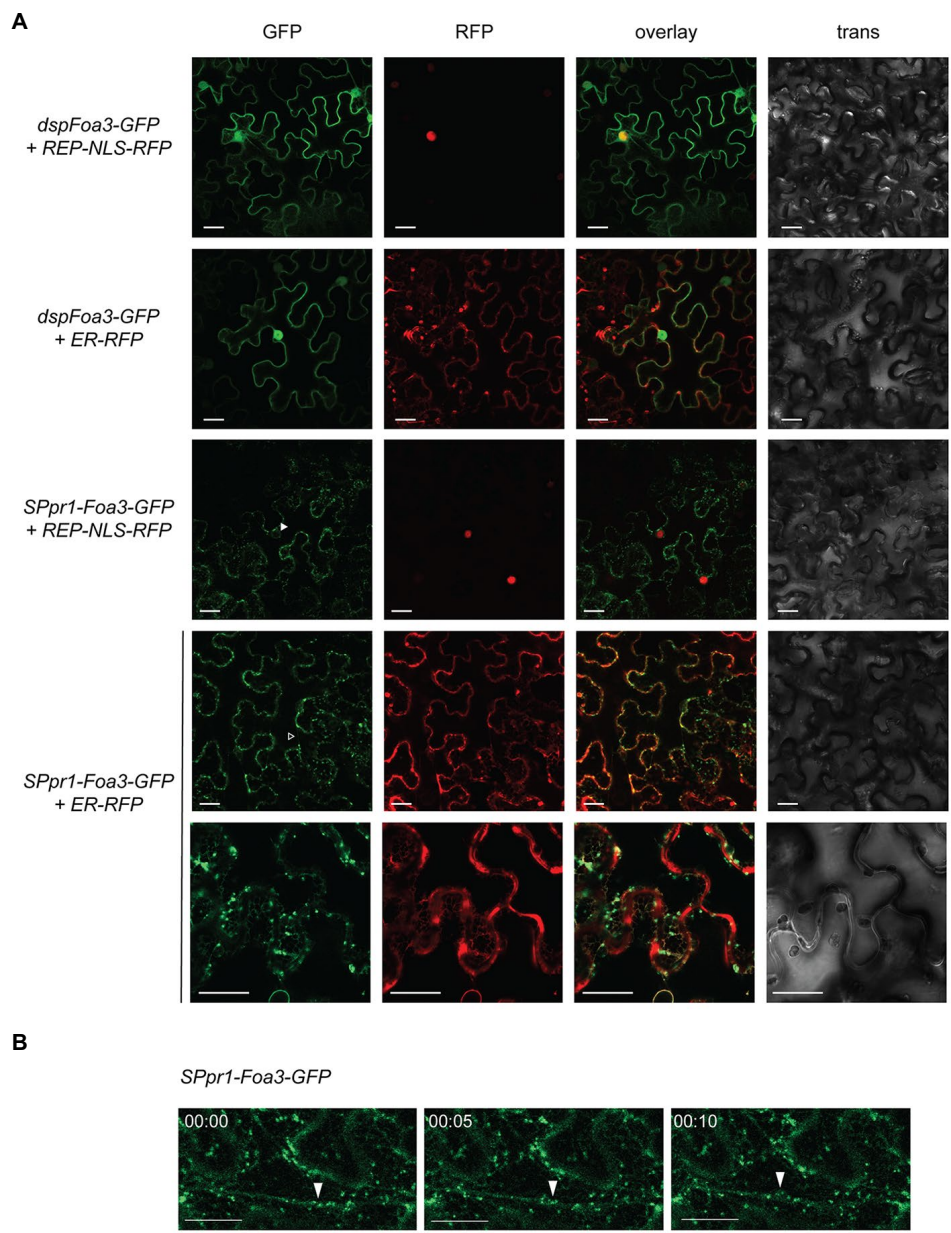
Subcellular localization of Foa3 produced with (sp) or without (dsp) a secretory signal peptide. **(A)** Confocal microscopy of Agro-infiltrated *Nicotiana benthamiana* leaves expressing the indicated constructs. Closed arrowhead indicates SPpr1-Foa3-GFP accumulating around a nucleus. Open arrowhead indicates SPpr1-Foa3-GFP at cytosolic strands. Size bars = 20 μm. **(B)** Time-lapse confocal images showing accumulation of SPpr1-Foa3-GFP in mobile subcellular structures. Closed arrows indicate Foa3-GFP labelled puncta moving along cytosolic strands.

Since both dspFoa3 and SPpr1-Foa3 efficiently suppressed defense responses despite being targeted to different locations, we set out to investigate their subcellular localization. First, *N. benthamiana* leaves expressing *dspFoa3-GFP* were subjected to laser scanning confocal microscopy. A GFP signal was revealed that is typical for a nuclear-cytosolic localization (Figure 2A). Indeed, co-expression with a nuclear marker (REP-NLS-RFP; Maio et al., 2019) confirmed accumulation in the nucleus, whereas only very little overlap with an endoplasmic reticulum (ER) marker was observed (Cao et al., 2018). In conclusion, expression of Foa3-GFP without a secretory signal peptide leads to its accumulation mainly in the cell nucleus and cytosol. This indicates that Foa3 exerts its PTI-suppressing activity at these subcellular locations. In contrast, expression of *SPpr1-Foa3-GFP* showed a very different localization pattern for the fluorescent effector protein. The GFP signal was predominantly observed in bright spots, most of which were mobile (Figure 2). A somewhat weaker signal was outlining the cell and was also visible around the nucleus and in cytosolic strands (Figure 2A). In addition, the GFP signal was detected in a net-like structure typical for the ER which indeed co-localized with the ER marker (Figure 2A). The bright punctate structures localized in the proximity of, but did not show a clear overlap with, the ER marker suggesting that they are not part of the ER. We did not detect a strong accumulation of SPpr1-Foa3-GFP in the cytosol or nucleus, even though it suppresses the PTI responses to an extent similar as dspFoa3-GFP. Since in a large fraction of the tagged effector the GFP was cleaved off, it is possible that mainly untagged Foa3 enters the cytosol and nucleus and is responsible for the observed PTI suppression. Altogether, SPpr1-Foa3-GFP seems to partially localize to the ER, but in addition it strongly accumulates in subcellular structures of unknown identity that can localize in physical proximity to the ER, but also move along cytosolic strands.

### Foa3 Can Deliver NS24 Into Host Cells

To provide additional evidence for intracellular translocation of SPpr1-Foa3, we monitored its ability to deliver an Avirulence protein to the cytosol. The non-structural movement protein (NSm) of tomato spotted wilt virus is recognized by the resistance protein Sw-5b, leading to an HR. Furthermore, it was shown that attaching a 21 amino acid peptide derived from NSm (NS21) to YFP is sufficient to physically bind to and activate Sw-5b (Zhu et al., 2017). As the interaction between NS21 and Sw-5b occurs intracellularly and produces an easily detectable cell death response, we predicted that attaching NS21 to Foa3 could serve as a proxy for its intracellular localization.

**Figure 3:**
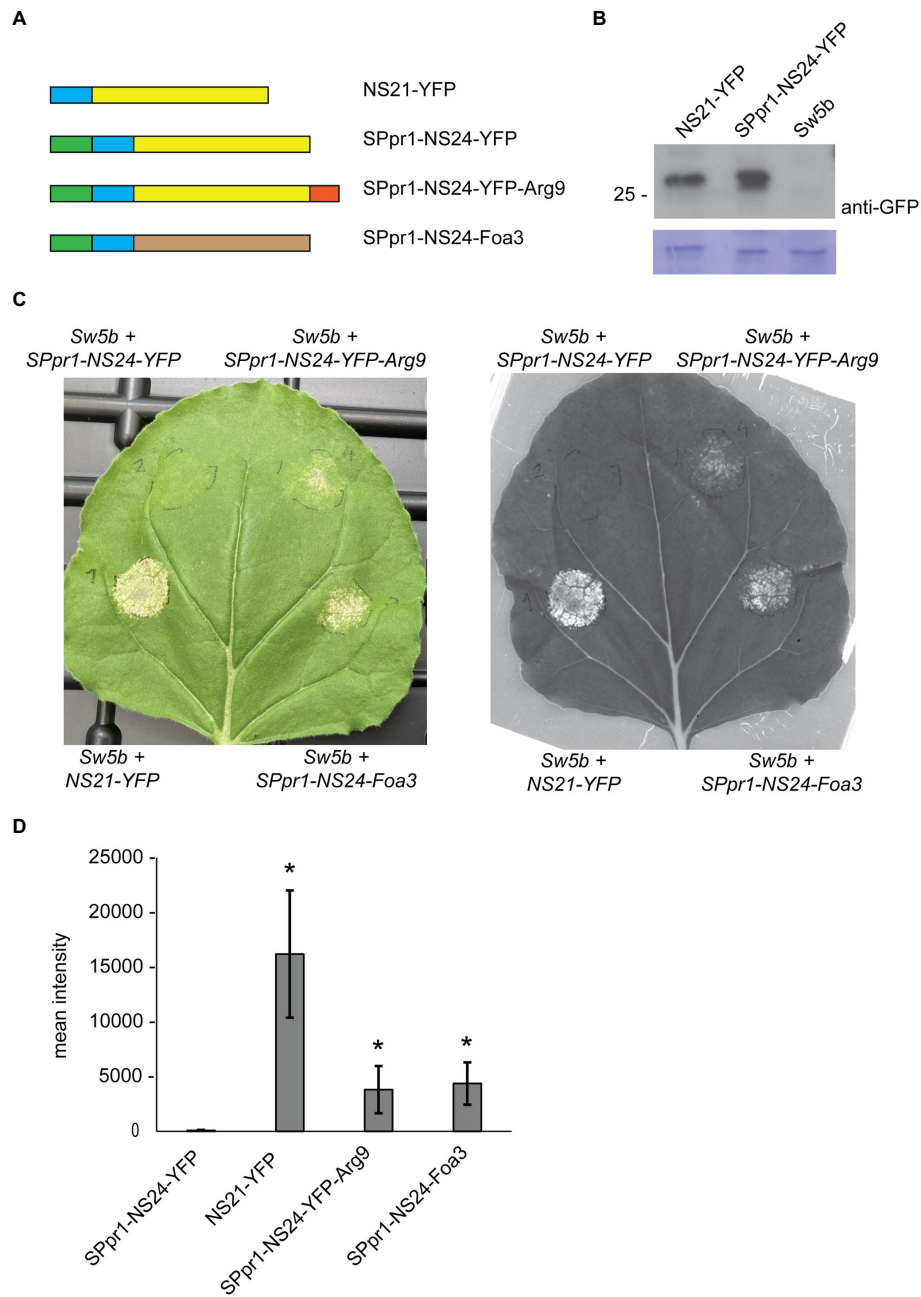
Foa3 can deliver an avirulence protein of tomato spotted wilt virus into host cells. **(A)** Schematic representation of the constructs used in *Nicotiana benthamiana* Agro-infiltration experiments. NS21/24 (blue), YFP (yellow), PR1 signal peptide (green), Arginine 9 (red) and Foa3 (brown) are shown. **(B)** Anti-YFP Western blot showing similar accumulation levels of NS21-YFP and SPpr1-NS24-YFP in Agro-infiltrated *Nicotiana benthamiana* leaves. Molecular weight (kDa) markers are shown on the left. Equal loading is verified by Coomassie staining of the blots. **(C)** *Sw-5b* triggered cell death in *Nicotiana benthamiana* leaves expressing the indicated constructs. A representative leaf is shown upon regular photography (left) or following red fluorescence imaging using a Chemidoc imager (right). Images were taken 5 days after Agro-infiltration. **(D)** Signal quantification of red fluorescence imaging of *Nicotiana benthamiana* leaves producing the indicated constructs. Statistically significant differences to the SPpr1-NS24-YFP construct are indicated (*p* < 0.05, *t*-test, *n* = 6 leaves). Experiments were repeated at least three times with similar results.

First, we investigated whether attaching the PR1 signal peptide to NS21-YFP would re-direct it to the secretory path and thus prevent intracellular accumulation and subsequent recognition by Sw-5b. To create a typical cleavage site in which the signal peptide sequence is followed by a set of polar amino acids, the NS21 unit was N-terminally extended by three amino acids, resulting in SPpr1-NS24-YFP (Figure 3A; section Materials and Methods). Zhu et al. established that N- or C-terminally extending the NS21 minimal unit does not interfere with its recognition by Sw-5b (Zhu et al., 2017). As reported earlier, co-expression of *NS21-YFP* with *Sw-5b* in *N. benthamiana* induced a strong cell death response (Figure 3C). In contrast, co-expression of *SPpr1-NS24-YFP* and *Sw-5b* did not produce a visible cell death response, indicating that secretion of NS24-YFP prevented its intracellular accumulation and thus the activation of the intracellularly localized Sw-5b resistance protein. Importantly, Western blotting showed that both NS21-YFP and SPpr1-NS24-YFP accumulated to similar levels, thus the lack of an HR was not due to poor stability or accumulation of the secreted NS24-YFP protein (Figure 3B). Instead, this result suggests that NS24-YFP lacks the ability to efficiently re-localize to the cytosol after being secreted.

Next, we tested whether attaching a cell penetrating peptide of nine consecutive arginine residues (Arg9) to NS24-YFP would result in its uptake, thereby triggering Sw-5b dependent cell death. Arg9-mediated uptake of target proteins has been demonstrated in different systems, among others in soybean and tomato (Dou et al., 2008; Numata et al., 2018). When the *SPpr1-NS24-YFP-Arg9* construct was co-expressed with Sw-5b, a cell death response was observed, indicating that NS24-YFP-Arg9 was successfully taken up by the plant cells. The cell death response was weaker than the one induced by cytoplasmic NS21-YFP but was readily detectable both by visual inspection as well as by monitoring red fluorescent emission as a proxy for cell death (Landeo Villanueva et al., 2021; Figure 3C). Next, we tested whether Foa3 could replace the function of Arg9 and mediate cellular entry of NS24. Thereto, SPpr1-NS24 was N-terminally fused to Foa3, and when this *SPpr1-NS24-Foa3* construct was co-expressed with *Sw-5b*, a clear cell death response was observed (Figure 3C). Quantification of red fluorescence showed that both SPpr1-NS24-YFP-Arg9 and SPpr1-NS24-Foa3 induced comparable levels of Sw-5b activation (Figure 3D). In conclusion, both Foa3 and Arg9 seem to enable cellular uptake of NS24 and thus activation of Sw-5b.

### Foa3 Can Inhibit PTI Upon Its Infiltration to the Apoplast

**Figure 4:**
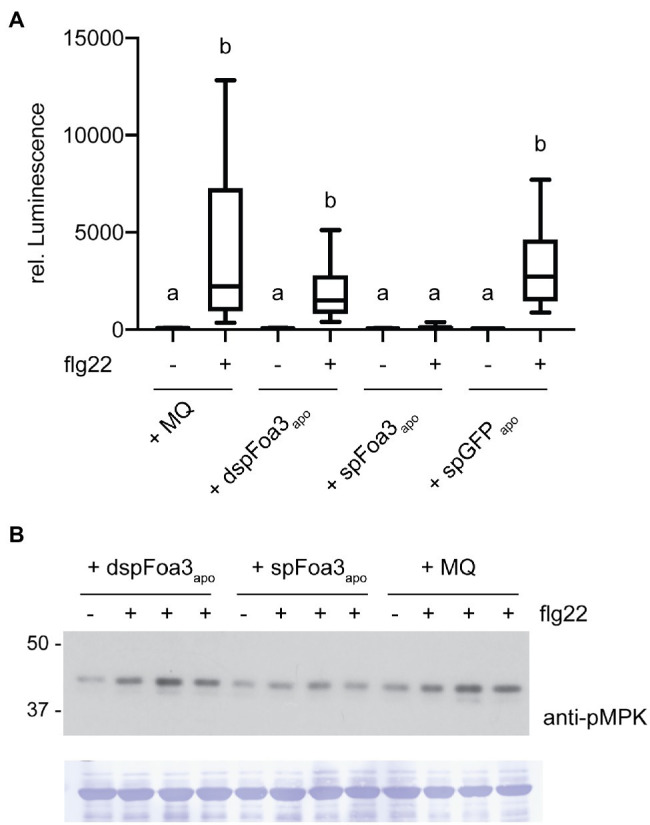
Infiltration of Foa3-containing apopastic fluid into *Nicotiana benthamiana* leaves results in suppression of flg22-triggered defense responses. **(A)** Flg22-triggered ROS burst in *Nicotiana benthamiana* leaves that were treated as follows: Infiltrated with water (MQ), infiltrated with apoplastic fluid isolated from *Nicotiana benthamiana* plants expressing either *dspFoa3-GFP* (dspFoa3-GFP_apo_) or *SPpr1-Foa3-GFP* (SPpr1-Foa3-GFP_apo_) or *SPpr1-GFP* (SPpr1-GFP_apo_), respectively. Boxes extend from the 25^th^ to the 75^th^ percentile, whiskers from the lowest to highest values, bar indicates the median, *n* = 16 leaf discs. Significant differences are indicated (*p* < 0.05, Kruskal-Wallace test). **(B)** Western blot showing flg22-triggered activation of MAP kinases in *Nicotiana benthamiana* leaves infiltrated with water (MQ) or apoplastic fluid isolated from *Nicotiana benthamiana* plants expressing either *dspFoa3-GFP* (dspFoa3-GFP_apo_) or *SPpr1-Foa3-GFP* (SPpr1-Foa3-GFP_apo_). Subsequently, leaves were treated with water (−) or flg22 (+) for 10 min. Three independent flg22-treated samples were loaded on blots next to each other. Molecular weight (kDa) markers are shown on the left. Equal loading is verified by Coomassie staining of the blots. Experiments were repeated three times with similar results.

Intracellular accumulation of SPpr1-Foa3-GFP could result from its uptake from the apoplast or from re-translocation (e.g., escape) out of the secretory pathway. To distinguish between these scenarios, we investigated whether infiltration of Foa3 into the apoplast would result in PTI suppression. Since *SPpr1-Foa3-GFP* expressing *N. benthamiana* accumulates Foa3 in the apoplast (Figure 1A), apoplastic fluid from these plants was collected and infiltrated into untreated plants. In addition, several controls were infiltrated: (1) apoplastic fluid from *dspFoa3-GFP* expressing plants, containing only trace amounts of Foa3-GFP; (2) apoplastic fluid of *SPpr1-GFP* expressing plants, containing high levels of GFP; and (3) MQ water. Subsequently, leaf discs obtained from the infiltrated plants were exposed to flg22 and ROS generation was measured. Plants that were infiltrated with either water or the apoplastic fluid from *dspFoa3-GFP* or *SPpr1-GFP* expressing plants showed a strong accumulation of flg22-triggered ROS (Figure 4A). In contrast, infiltration of the apoplastic fluid that contained high amounts of Foa3-GFP resulted in an almost complete suppression of the flg22-triggered ROS response (Figure 4A). These results could imply that Foa3 is taken up from the apoplast, reaching its intracellular localization where it suppresses flg22-triggered ROS generation.

To further validate these findings, we monitored flg22-triggered activation of MAPKs in *N. benthamiana* plants that were treated similarly as for the ROS assay. Infiltration of water or apoplastic fluid from a *dspFoa3-GFP* expressing plant resulted in MAPK activation as revealed by detection of phosphorylated MAPKs by Western blotting (Figure 4B). In contrast, the infiltration of SPpr1-Foa3-GFP containing apoplastic fluid resulted in much lower levels of activated MAPKs (Figure 4B). In this experiment, predominantly the upper MAPK band was detected, which corresponds to SIPK (SALICYLIC ACID-INDUCED PROTEIN KINASE; Segonzac et al., 2011). This difference may be due to the presence of Agrobacteria in the experimental setup of Figure 1, and absence thereof in Figure 4. Nonetheless, only the treatment with Foa3-containing apoplastic fluid led to suppression of the flg22-triggered accumulation of activated MAPKs. Together with the observed suppression of ROS generation by apoplastically delivered Foa3, these findings point to effector uptake by plant cells from the apoplast.

## Discussion

In this study we demonstrate that, when produced *in planta* without a secretory signal peptide, Foa3 efficiently suppresses flg22-triggered ROS accumulation and -MAPK activation, indicating that it targets an intracellular host factor required for early flg22 signaling. Since chitin-triggered ROS accumulation was suppressed as well (Tintor et al., 2020), Foa3 apparently intercepts signaling triggered by multiple pattern recognition receptors. As dspFoa3-GFP shows a nucleo-cytosolic accumulation, it is likely that its PTI suppressing activity is carried out at these subcellular locations. Interestingly, suppression of the same PTI responses was observed when Foa3 was targeted for secretion *via* the PR1 signal peptide or when the effector was directly infiltrated into the apoplast. Two scenarios could explain this finding. In the first one, Foa3 is taken up from the apoplast and subsequently translocated to the nucleo-cytoplasmic compartment where it exerts a virulence activity. In the second scenario, Foa3 would have to act on two different targets, resulting in both cases in almost complete inhibition of flg22 and chitin-signaling. Even though effectors can have multiple targets, these are generally present at the same location and/or affect different processes. To our knowledge, an effector targeting the same process *via* separate intracellular and extracellular functions has not yet been described, making the latter possibility less likely.

Besides its PTI-suppressing activity, Foa3 successfully delivered a secreted NSm24 avirulence peptide to the matching intracellular Sw-5b resistance protein, resulting in a cell death response. In contrast, when an NSm24-YFP fusion protein was targeted for secretion, it did not induce a Sw-5b-triggered cell death, indicating that YFP itself was not able to translocate to the cytosol. To exclude whether the observed Sw-5-specific cell death could be due to escape of the NSm24-Foa3, but not the NSm24-YFP protein, from the secretory pathway upon Agro-infiltration, apoplastic fluid isolated from wildtype *N. benthamiana* leaves Agro-infiltrated with *NSm21-YFP*, *SPpr1-NSm24-YFP*, *SPpr1-NSm24-YFP-Arg9* and *SPpr1-NSm24-Foa3* was infiltrated in *Sw5b* expressing *N. benthamiana* leaves (data not shown). No *Sw5b*-specific cell death was observed following infiltration of either the NSm24-Foa3 fusion or the positive control NSm24-YFP-Arg9. The same constructs induced cell death when expressed in *Sw5b* plants, showing their functionality (Figure 3). The lack of Sw5b-induced cell death following apoplastic fluid infiltration is likely due to proteolytic activity in the extracellular spaces resulting in cleavage of the NSm24 peptide from the Foa3 effector protein and from the Arg9 peptide. It is well established that the extracellular spaces from Solanaceous species contain potent proteases that remove (affinity) tags from effector proteins secreted to the apoplast (van Esse et al., 2006) as also can be seen in Figure 1A. Cleavage could have occurred at any step between agroinfiltration, harvesting of the apoplastic fluid or following infiltration, making the latter experiment inconclusive. Potential uptake of the Foa3 protein from the apoplastic space could occur directly from the apoplast into the cytosol, or *via* retrograde transport from the ER following its internalisation by endocytosis and/or following escape from endosomal vesicles. Future experiments using *in situ* immune labeling could reveal whether there is a preferred uptake mechanism, or whether these processes occur simultaneously. Notably, when expressed *in planta* with a secretory signal peptide, numerous fungal and oomycete effector also appear to localize intracellularly, mostly demonstrated by an intracellular avirulence activity (Rehmany et al., 2005; Bos et al., 2006; Rafiqi et al., 2010; Blondeau et al., 2015). However, besides the *P. nodorum* effector ToxA (Liu et al., 2009), in these cases it was not tested whether the avirulence activity was also observed after infiltration of these effectors into the apoplast, as shown here.

When expressed without a signal peptide Foa3 accumulates in the cytosol and nucleus, strongly suggesting that it acts at these locations to inhibit PTI signaling. In contrast, the GFP fluorescence of secreted Foa3 showed a relatively weak signal in the cytosol and ER but was strongly detected in punctate structures that localized close to the ER, but also moved along cytosolic strands. It is possible that these structures represent vesicles containing proteins targeted for secretion. However, expression of secreted GFP did not produce similar bright fluorescent structures (Supplementary Figure 2). This could indicate that Foa3 accumulates at an intermediate step of the secretory path, even though it eventually reaches the apoplast, since it is present in the apoplastic fluid (Figure 1A). Interestingly, several host-translocated effectors showed similar concentrated accumulation when expressed *in planta* with a signal peptide (Kloppholz et al., 2011; Petre et al., 2016). In contrast, other intracellular effectors showed a more uniform distribution, indicating that this localization pattern is effector-specific (Rafiqi et al., 2010). It is unlikely that this localization is merely a consequence of overexpression since dspFoa3-GFP accumulates to a similar level but does not exert the same localization pattern. However, whether the punctate localization structures in which Foa3 accumulate are functionally correlated with its ability to translocate into host cells is at present unclear. Additional cell biological studies are required to elucidate the identity and role of these subcellular structures.

Whether a potential cell entry of Foa3 is mediated *via* binding to a cell surface structure such as (glycol) lipids is currently unknown and would require experimental testing. Our data indicate that Foa3 mediates uptake of NS24 as efficiently as the known cell entry peptide Arginine 9. The precise working mechanism of Arginine 9 is not understood, but it is thought that positively charged arginine residues electrostatically bind to negatively charged cell-surface molecules, possibly inducing endocytosis of the target protein (Duchardt et al., 2007; Schmidt et al., 2010). Foa3 itself carries six arginine and two lysine residues, which are not clustered in a protein structure fold prediction by AlphaFold, arguing against localized strong positive surface charges. However, several positively charged amino acids are predicted to be surface-localized, theoretically enabling an Arginine 9-like, charge-based uptake mechanism of Foa3. The supposed uptake of Foa3 and its strong PTI-suppressing activity provide an ideal tool to investigate the underlying cell biological processes.

## Data Availability Statement

The original contributions presented in the study are included in the article/Supplementary Material, further inquiries can be directed to the corresponding author.

## Author Contributions

NT, IB, and GN performed the experiments. NT and FT designed the project. NT wrote the paper with help of FT. All authors contributed to the article and approved the submitted version.

## Funding

FT and NT obtained support from the NWO-Earth and Life Sciences funded VICI Project No. 865.14.003 and from ENZA Zaden.

## Conflict of Interest

The authors declare that the research was conducted in the absence of any commercial or financial relationships that could be construed as a potential conflict of interest.

## Publisher’s Note

All claims expressed in this article are solely those of the authors and do not necessarily represent those of their affiliated organizations, or those of the publisher, the editors and the reviewers. Any product that may be evaluated in this article, or claim that may be made by its manufacturer, is not guaranteed or endorsed by the publisher.

## Supplementary Material

Supplementary Figure 1 Plasmid maps of pBIN61 containing the cassettes *SPpr1-NSm24-YFP*, *SPpr1-NSm24-YFP-Arg9* and *SPpr1-NSm24-FOA3* **(A)**, as well as pGWB-*dspFOA3-GFP* and pGWB451-*SPpr1-FOA3-GFP* **(B)**.

Supplementary Figure 2 Confocal microscopy of Agro-infiltrated *N. benthamiana* leaves expressing *SPpr1-GFP*, hence producing secreted GFP. The bright punctate structures observed with *SPpr1-Foa3-GFP* were not observed upon *SPpr1-GFP* expression.
