# Supplementary figures and images for: The Intracellularly Acting Effector Foa3 Suppresses Defense Responses When Infiltrated Into the Apoplast
Source: Front Plant Sci. 2022 May 23;13:813181. doi: 10.3389/fpls.2022.813181 (PMC9169155; doi:10.3389/fpls.2022.813181)

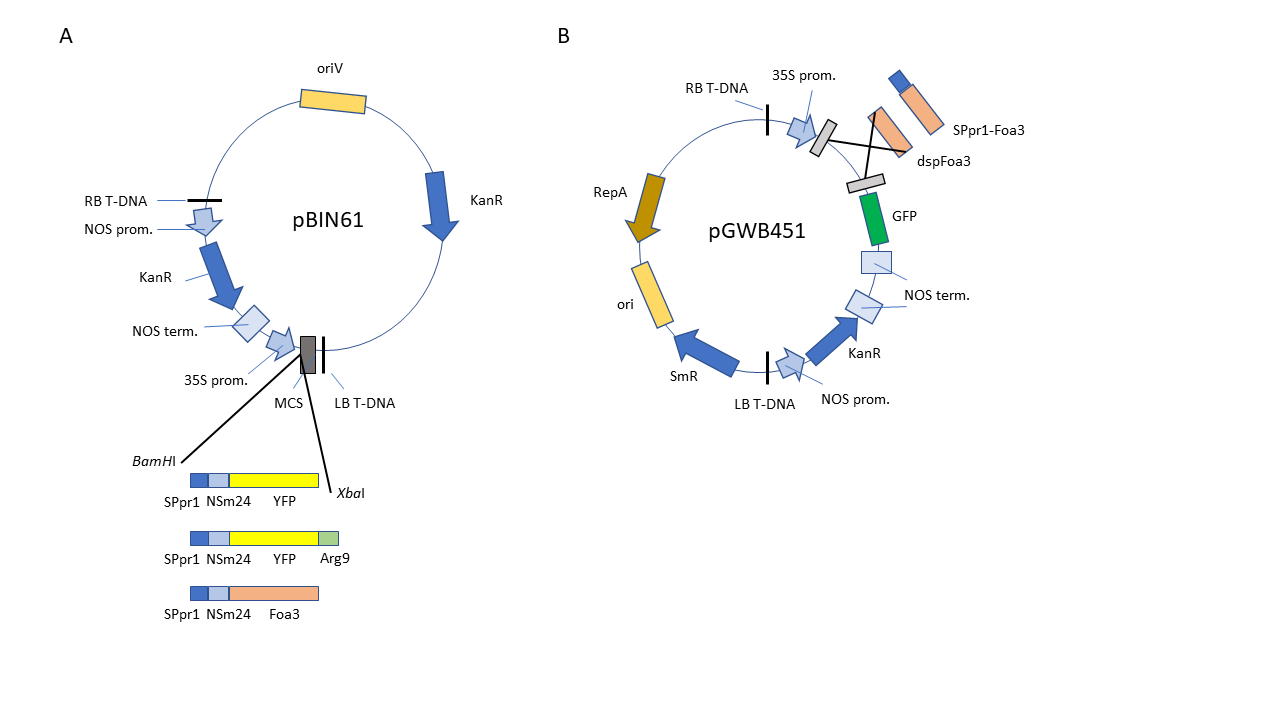

Supplement: Supplementary Figure 1 — Plasmid maps of pBIN61 containing the cassettes SPpr1-NSm24-YFP, SPpr1-NSm24-YFP-Arg9 and SPpr1-NSm24-FOA3 (A), as well as pGWB-dspFOA3-GFP and pGWB451-SPpr1-FOA3-GFP (B). [file Image_1.TIF]

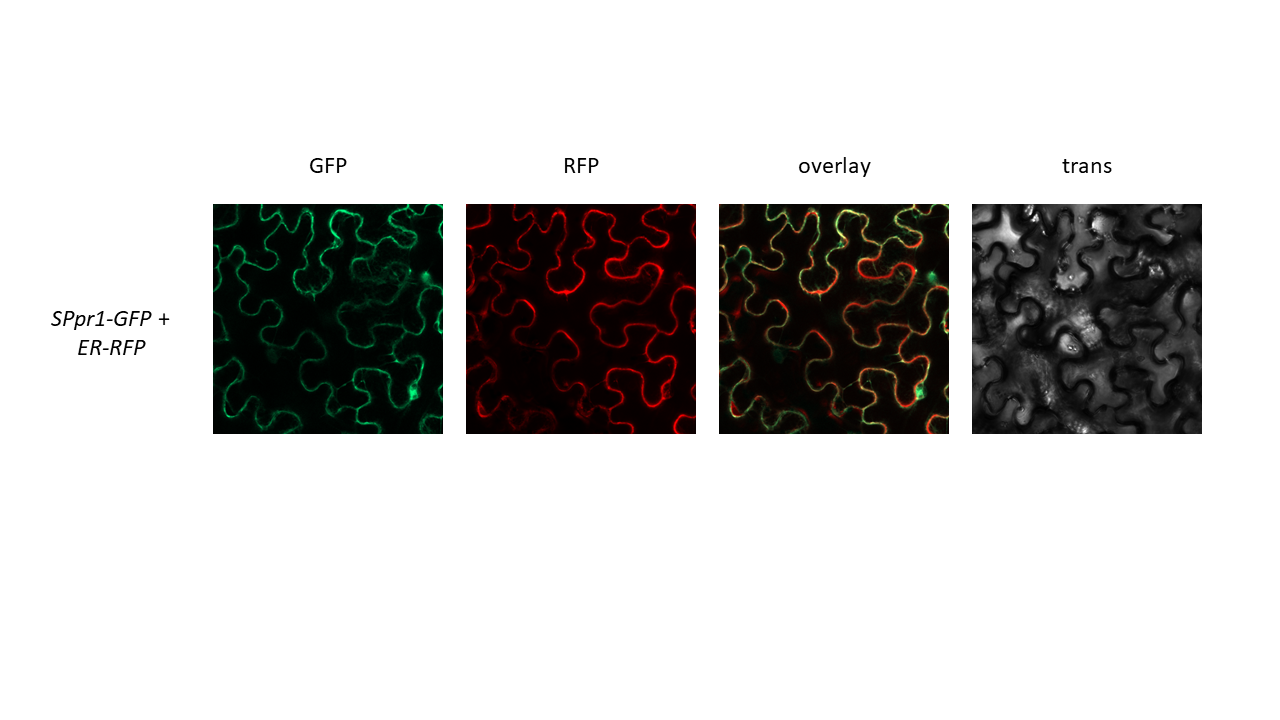

Supplement: Supplementary Figure 2 — Confocal microscopy of Agro-infiltrated N. benthamiana leaves expressing SPpr1-GFP, hence producing secreted GFP. The bright punctate structures observed with SPpr1-Foa3-GFP were not observed upon SPpr1-GFP expression. [file Image_2.TIF]
